# Protease-activated receptor 2 signaling modulates susceptibility of colonic epithelium to injury through stabilization of YAP in vivo

**DOI:** 10.1038/s41419-018-0995-x

**Published:** 2018-09-20

**Authors:** Longmei He, Yiming Ma, Weiwei Li, Wenxiao Han, Xinhua Zhao, Hongying Wang

**Affiliations:** State Key Laboratory of Molecular Oncology, National Cancer Center/National Clinical Research Center for Cancer/Cancer Hospital, Chinese Academy of Medical Sciences, Peking Union Medical College, Beijing, China 100021

## Abstract

Hippo signaling plays critical roles in intestinal regeneration. However, the mechanisms which regulate its activity in vivo are largely unknown. We hypothesize that protease-activated receptor 2 (PAR2) signaling, which could be activated by trypsin, might affect YAP activity in the setting of tissue damage and regeneration. It is found that knockout of PAR2 severely aggravates the mucosal damage induced by dextran sodium sulfate (DSS) in mouse, which correlated with notable repression of YAP protein in colonic epithelial cells. Although the cytokine expression is reduced, the damage of colonic crypt is more severe after DSS-induced colitis in PAR2-/- mouse. In vitro, PAR2 activation causes the accumulation of YAP, while knockdown of PAR2 with shRNA dramatically represses the expression of YAP protein in different intestinal epithelial cell lines. Moreover, forced expression of YAP significantly reduces the production of reactive oxygen species (ROS) and the sensitivity to nitric oxide-induced apoptosis in PAR2-deficient condition. Further studies show that PAR2 signaling stabilizes YAP protein but independent of Lats. Nevertheless PAR2 activation increased the binding of YAP with protein phosphatase PP1. Inhibition of PP1 with specific siRNA blocked PAR2-induced dephosphorylation of YAP. Taken together, PAR2 signaling might modulate susceptibility of colonic epithelium to injury through stabilization of YAP.

## Introduction

The precise control of organ size is crucial during animal development and tissue regeneration. The uncontrolled overgrowth of tissue results in the formation of tumors. Chronic inflammation is a repetitive process of damage and repair, and has been shown to promote carcinogenesis in different organs. Deregulation of tissue regeneration after tissue damage contributes to inflammation-related carcinogenesis. Therefore, understanding the mechanisms which control the regeneration is critical. Recently, emerging evidence showed that Hippo signaling plays an important role in organ size control and tissue regeneration. As the key molecule of the Hippo pathway, YAP is known to be regulated by serine/threonine kinase Lats1/2 culminating in phosphorylation of YAP at serine 127 (S127) and cytoplasmic sequestration^1^. Nuclear YAP binds to TEAD and stimulates the transcription of target genes such as CTGF, CYR61, and AREG^2^. YAP is involved in stem cell biology and plays important roles in the homeostasis, regeneration, and tumorigenesis of gut^3^. Although accumulation of YAP is related to overgrowth of organs and tumorigenesis, the mechanism by which its activity is regulated during tissue regeneration is largely unknown.

In the gut, proteases play critical roles during the pathological processes of trauma, inflammation, and tumorigenesis. Besides the proteases produced by inflammatory cells, a large amount of proteases derived from host cells and bacteria are enriched in intestinal lumen, an example being trypsin^4^. Excessive release of proteases has been reported in functional and inflammatory bowel diseases^5^. Dysregulation or interruption of epithelial barrier function leads to the exposure of intestinal epithelial cells (IECs) to luminal content. Importantly, some proteases selectively cleave and activate protease-activated receptors (PARs), which are G protein-coupled receptors and expressed widely in the epithelium of the gut^6^. The PARs family consists of four members (PAR 1–4) and PAR2 is the cardinal one activated by trypsin^6^. Therefore, PARs are likely reasonable candidates to sense mucosal tissue injury and initiate or regulate a response of repair and regeneration in the gut mucosa. Most recently, PAR2 has been shown to participate in the regeneration of various organs. Mice lacking PAR2 exhibit deregulation of tissue regeneration after injury in the pancreas, liver, and digits^7^. However, the underlying mechanism is not known. In this study, we tested the hypothesis that PAR2 signaling regulates the colonic mucosal regeneration through YAP after injury.

## Materials and methods

### Animal studies

C57BL/6 mice were purchased from Beijing Vital River Laboratory Animal Technology Co. Ltd (Beijing, China). PAR2 knock-out mice (B6.Cg-F2rl^1*tm*1M*slb*^/J mice) were obtained from the Jackson Laboratory (CA, USA). Six to eight-week-old male mice were used for all experiments. Mice were housed under controlled conditions (25–27 °C, 45–55% humidity, and 12-h day/night cycle). The study protocol was approved by the Committee of Animal Experimentation, Cancer Hospital, Chinese Academy of Medical Sciences.

Dextran sulfate sodium (DSS)-induced colitis mouse model was established as described previously^8^. Mice received 2.5% DSS (MW 36,000–50,000 kDa, MP Biomedical) via drinking water for 5 days, followed with regular water without DSS. Mice were killed after 2 days (DSS + 2d) or 4 days (DSS + 4d).

The histological evaluation of crypt damage was performed. The evaluation grade is dependent on crypt morphology, 0: no damage; 1: basal 1 /3 crypt damaged; 2: basal 2 /3 crypt damaged; 3: only surface intact; 4: entire crypt and surface loss. Data are represented as the percentage of mice per group with the indicated score. The evaluation was performed by a pathologist blinded to the animal groups.

### Studies in cultured cells

All cell lines used in the study were purchased from ATCC (Manassas, VA, USA). HT-29, SW620, HEK293, and A549 cells were cultured in Dulbecco’s modified Eagle’s medium/F12 (Hyclone, UT, USA) supplemented with 10% FBS (Gemini Bio, CA, USA), 1% penicillin and streptomycin. HEK293T cells were cultured in DMEM supplemented with 10% FBS, penicillin, and streptomycin. For chemical reagent treatment, cells were incubated in DMEM/F12 without supplements for 12 h and then treated with the PAR2-selective activating peptide (SLIGRL-NH2) (Shanghai Apeptide Co. Ltd, China) or the phosphatase inhibitor OA (Okadaic acid, Cell Signaling) for different times.

### Virus infection and transfection of siRNA

Stable transfectant cell lines with PAR2 knockdown were enriched by puromycin according to the protocol described previously^9^. Human wild-type or mutant YAP1 CDS were cloned and inserted into pLVX-IRES-NEO plasmid, and lentivirus was packaged in HEK293T cells by using pLVX-IRES-Neo system as reported before^10^. Forty-eight hours after infection, YAP1-overexpressed lentiviral supernatant was filtered through a 0.45-µm filter and used to infect HT-29-shCtrl and HT-29–shPAR2 cells. Cells were selected with 300 µg/mL G418 in the culture medium 48 h after infection.

Cultured cells were transient transfected with siRNA using Lipofectamine 2000 (Invitrogen, CA, USA). Different treatment of cells were carried out 24 h after transfection. The sequences of siRNA targeting PP1 were as follows: siRNA (#1, 5′-CCGAGAGCAACUACCUCUUTT-3′, #2, 5′-GCAGUCUAUGGAGCAGAUUTT-3′) and negative control (GenePharma, Suzhou, China).

### Flow cytometry analysis of cell apoptosis

HT-29 stably transfected cells were treated with 0.5 mM DETA NONOate (Cayman Chemical) for 48 h. The floating and adherent cells were harvested for the analysis of apoptosis. Cells (10^6^/mL) were incubated with 5-μl Annexin V–FITC (FITC Annexin V Apoptosis Detection Kit II, BD Biosciences, 556570) at room temperature for 20 min in the dark. After the incubation with Propidium Iodide (FITC Annexin V Apoptosis Detection Kit II), the cells were analyzed using flow cytometry.

### Colony formation and 3D cell culture

Cells were seeded at low density (300 cells/well of 12-well plate) and allowed to grow till visible colonies appeared. The cells were then stained with crystal violet solution and colonies were counted.

For 3D cell cultures, Type 1 collagen (Advanced Biomatrix, CA, USA) was diluted at 2 mg/mL in DMEM/F-12 containing 10% FBS. Three collagen layers were set up in 12-well culture plates, with the middle layer containing 5000 single cells. A total of 400-μL medium was added on top and changed every 2–3 days. Colonies were observed and counted after 10 days.

### Detection of ROS level

Level of intracellular ROS was measured with the ROS assay kit (Beyotime Institute of Biotechnology, Shanghai, China). Briefly, cells were treated with fluorescent probes DCFH-DA (1:1000) and incubated at 37℃ for 20 min. Cells were incubated with 1 mM H_2_O_2_ for 2 h and then washed with PBS for three times. The fluorescence intensity was measured by flow cytometry.

### mRNA expression profiling and data analysis

Total RNA containing small RNA was extracted from stable transfectant HT-29 cells by using Trizol reagent (Invitrogen) and purified with mirVana miRNA Isolation Kit (Ambion, Austin, TX, USA). The Agilent 8 × 60 K mRNA microarray was constructed at CapitalBio Corporation (Beijing, China). The array data were analyzed for data summarization, normalization, and quality control by using the GeneSpring software V13 (Agilent). To select the differentially expressed genes, we used threshold values of ≥ 2 and ≤ −2-fold change, and Benjamini–Hochberg corrected *p*-value ≤ 0.05.

### Immunohistochemistry

Mouse colonic samples were embedded in paraffin. Four-millimeter sections were cut and stained with hematoxylin and eosin (H&E) or used for immunohistochemical analysis. Staining experiments were repeated on individual tissue sections prepared from separate mice. The primary antibody used in immunostaining was rabbit anti-YAP (Abcam, ab39361). The secondary antibody used in immunostaining was two-step plus®Poly-HRP Anti-Mouse/Rabbit IgG Detection System (OriGene, Wuxi, China). Sections were counterstained with hematoxylin (ZSGB-Bio, Beijing, China, ZLI-9610).

### Western blotting

Cultured cells were lysed by RIPA buffer (Applygen Technologies Inc, Beijing, China) with proteinase inhibitor cocktail (Roche, Mannheim, Germany) and protein phosphatases inhibitor complex (Applygen). The extracted proteins were quantified using a protein assay kit (Applygen). Equal amounts of protein from each sample were fractionated by 10% SDS-PAGE gel and transferred to polyvinylidene difluoride membranes. The western blots were performed with primary antibodies to phospho-Lats1 (S909, Cell Signaling, #9157), phospho-YAP (S127, Cell Signaling, #13008), phospho-YAP (S397, Cell Signaling, #13619), Lats1 (Cell Signaling, #3477), YAP (Cell Signaling, #4912), PP2A Antibody Sampler Kit (Cell Signaling, #9780), PAR2 (Abcam, ab180953), β-actin (Sigma-Aldrich, MO, USA), and PP1 (Santa Cruz, sc-7482). Nuclear and cytosol fractions were prepared by using Nuclear-Cytosol Extraction Kit (APPLYGEN, #P1200), as described by the manufacturer. The interest proteins YAP, HSP90 (Abcam, ab133491, cytosol control), and PARP (Cell Signaling, #9542, nuclear control) were detected by western blotting. Signals were quantified by ImageQuant™ LAS 4000 image-forming system.

### Immunoprecipitation

Protein extracts from cultured cells were prepared using immunoprecipitation lysis buffer (Thermo, IL, USA, #87787) with proteinase inhibitor cocktail (Roche) and protein phosphatases inhibitor complex (Applygen). The lysates were centrifuged for 10 min at 13,000 rpm at 4 ℃, and supernatants was incubated with anti-YAP1 antibody (Bethyl Laboratories, Inc, TX, USA) or IgG (Santa Cruz, sc-2027) for 2 h, followed by incubation with protein A/G agarose beads (Santa Cruz, sc-2003) overnight. The beads were washed using PBS buffer for three times, and then boiled in SDS-loading buffer. Targeted proteins were detected with western blotting.

### RNA isolation and real-time PCR

Total RNA was isolated from cells by using Trizol reagent (Invitrogen). RNA was treated with DNase I and reverse transcribed using a transcription kit (Thermo, Vilnius, Lithuania, K1642) to get the total cDNA as templates for real-time PCR detection. Real-time PCR was performed using SYBR premix Ex Taq II (TaKaRa Bio Inc., Dalian, China) on a Bio-Rad CFX96 machine. The primers used for real-time PCR were shown in Supplementary Table 1.

### Statistical analysis

Statistical analyses were performed on data collected from at least three independent experiments. Data were analyzed using GraphPad Prism5 software and presented as means ± SD. Comparison of the two groups was made using Student’s *t*-test for unpaired data. The difference was considered statistically significant when *p*-value was <0.05.

## Results

### PAR2 deficiency impairs regeneration of colonic mucosa following DSS-induced injury in mice

**Fig. 1:**
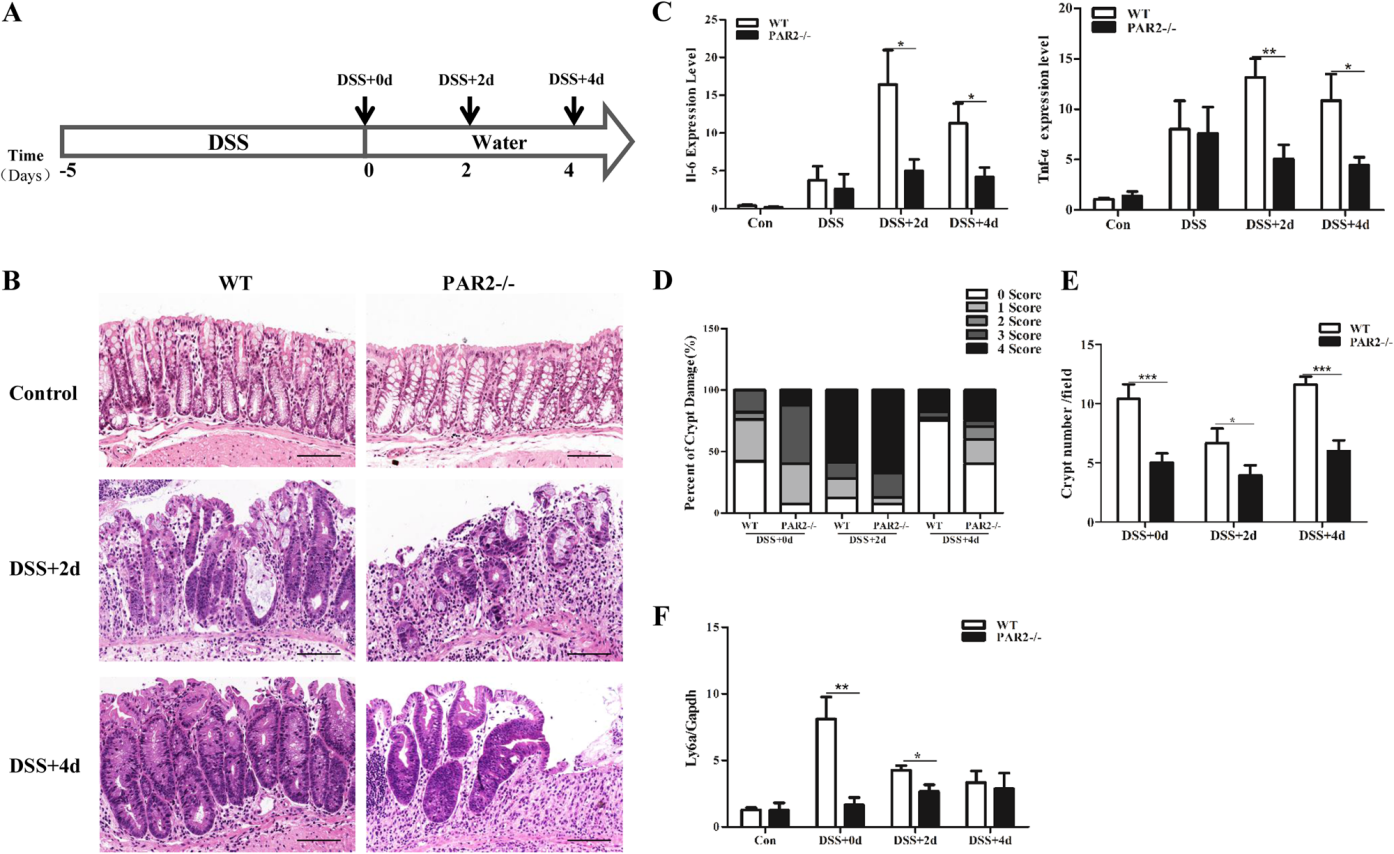
PAR2 deficiency impairs colonic regeneration following DSS-induced injury in mouse. **a** Schematic overview of DSS-induced colitis model in mouse. **b** H&E staining of colon sections taken from the mice 2 (DSS + 2d) or 4 days (DSS + 4d) after the administration of DSS. Scale bars represent 100 μm. **c** Expression of IL-6 and TNF-α mRNA were measured by real-time PCR with WT and PAR2^-/-^mouse colon. **d** Depth of crypt damage was assessed and shown (0, no damage; 1, basal 1/3; 2, basal 2/3; 3, only surface intact; and 4, entire crypt and surface loss). **e** Quantification of crypt number from five consecutive fields of each group. Data are shown as crypt number per field (magnification, × 250). **f** Expression of Ly6a mRNA was measured by real-time PCR with WT and PAR2-/- mouse colon. Data are shown as mean ± SEM. N.S., no significance; *p < 0.05; **p < 0.01; ***p < 0.001 vs. corresponding control

To test whether PAR2 signaling participates in colonic mucosal regeneration after injury, DSS-induced colitis and regeneration model were used (Fig. 1a). After the 5-day DSS treatment, mice show loss of body weight, diarrhea, and bloody stool. There was no significant difference on general symptoms between wild-type (WT) and PAR2 knock-out mice (PAR2^-/-^). H&E staining shows that inflammation of colonic mucosa was induced in both WT and PAR2^-/-^ mice (Fig. 1b, Figure S1). Consistent with previous reports^11^, the cytokine expression after DSS treatment declined in PAR2^-/-^ mice (Fig. 1c). However, the damage of crypt is more severe (Fig. 1d), and the number of crypts significantly decreased in PAR2^-/-^ mice (Fig. 1e). Furthermore, the expression of Ly6a, a marker related to regenerating crypt^12^, is dramatically repressed in PAR2^-/-^ mouse (Fig. 1f). The data implies that deficiency of PAR2 signaling sensitizes colonic epithelium to DSS-induced damage and retards epithelial regeneration in mice.

### Deficiency of PAR2 reduces YAP protein both in vivo and in vitro

**Fig. 2:**
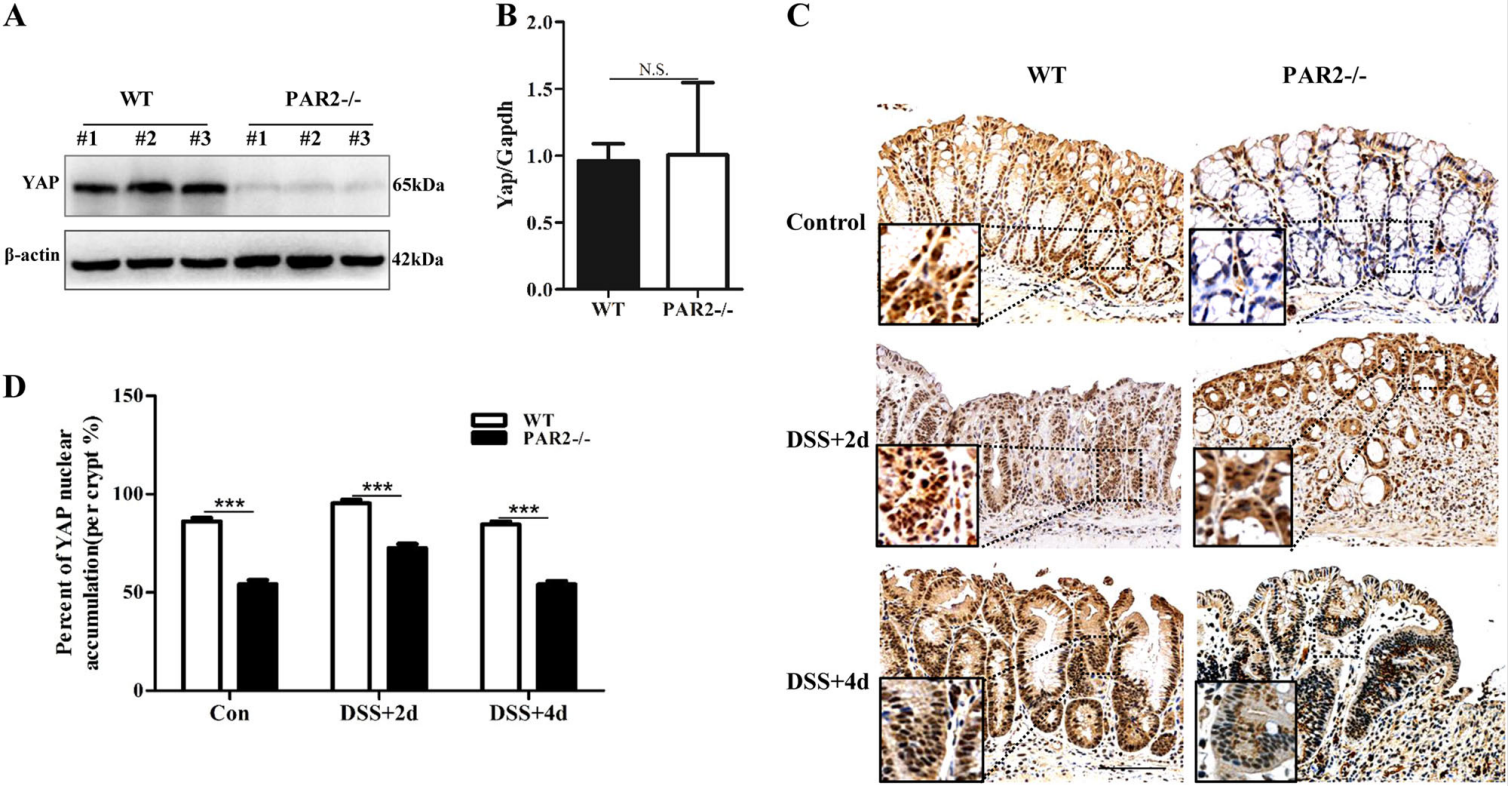
Knockout of PAR2 reduced YAP protein in vivo. **a** Protein and (**b**) mRNA expression of YAP in mouse colon was detected with western blotting or RT-PCR, respectively. **c** Immunohistochemical analysis of paraffin-embedded colon sections from wild-type and PAR2 knock-out mice. Scale bars represent 100 μm. **d** Number of colon epithelial cell with nuclear YAP was quantified. Data are shown as mean ± SEM. N.S., no significance; ***p < 0.001 vs. corresponding control

Given the emerging role of YAP in mucosal regeneration, YAP expression was examined in the setting of knockout of PAR2 expression. YAP expression of colon tissue is dramatically reduced at the protein level (Fig. 2a) without any effect on the mRNA level (Fig. 2b) in PAR2^-/-^ mice. Immunochemistry staining of YAP shows the decrease of YAP nuclear accumulation in most colonic epitheliums of PAR2^-/-^ mice (Fig. 2c) (Figure S2). In addition, the upregulation of YAP protein at 2 or 4 days after DSS treatment was reduced in PAR2^-/-^ mice, especially at 4 days (Fig. 2c, d).

**Fig. 3:**
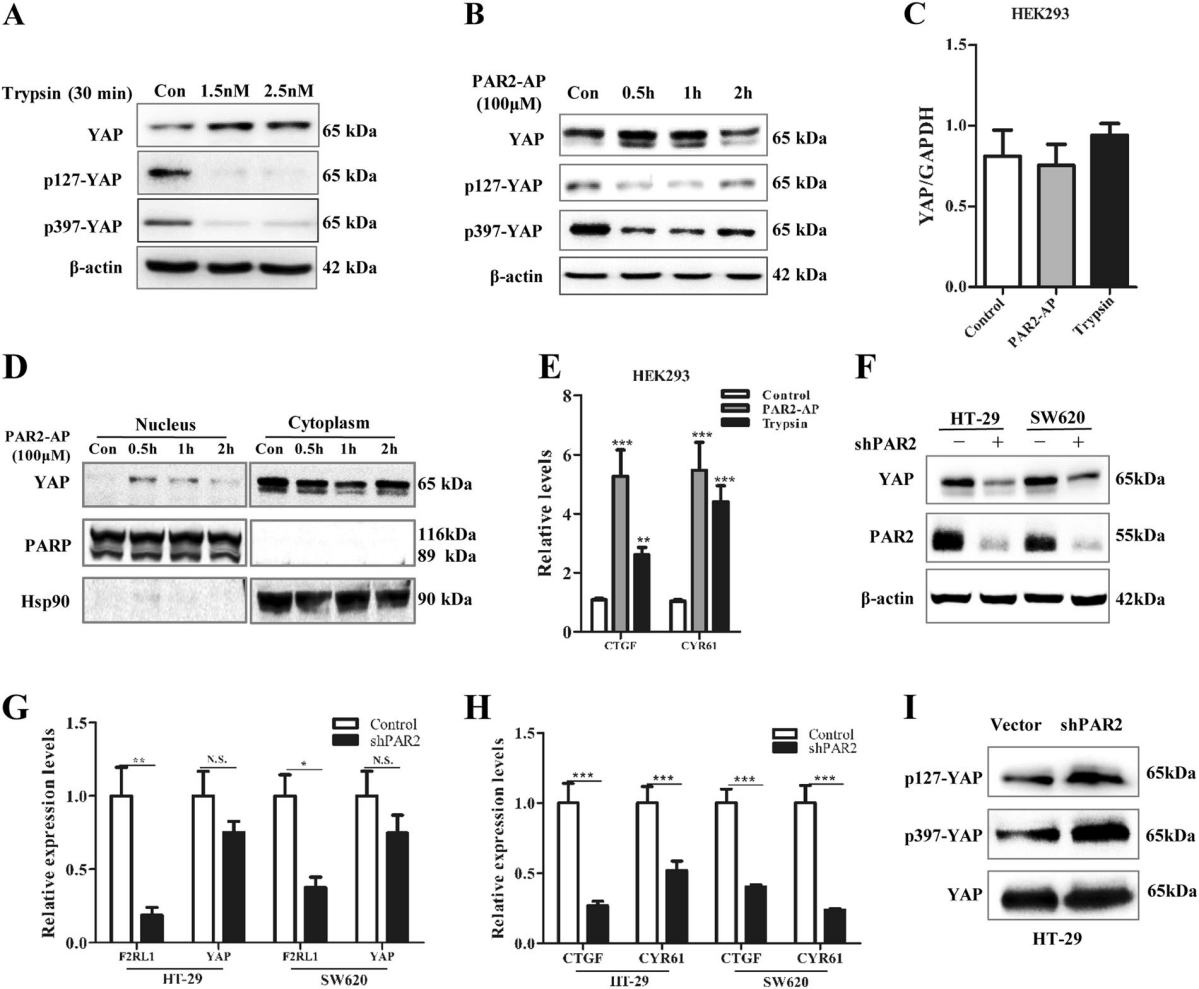
Activation of PAR2 promotes YAP stability. HEK293 cells were serum-starved for 12 h and then stimulated with (**a**) trypsin (1.5 nM or 2.5 nM) or (**b**) PAR2-activating peptide (AP) (100 μM) for different times. The level of total YAP, p127-YAP, and p397-YAP were measured with western blot. **c** HEK293 cells were serum-starved for 12 h and then stimulated with PAR2-AP (100 μM) or trypsin (2.5 nM) for 1 h. Expression of YAP mRNA was measured with real-time PCR. **d** Western blot was used to measure YAP protein expression in the nuclear and cytoplasmic fractions from HEK293 cell after PAR2 activation for different time. PARP (nuclear marker) and Hsp90 (cytoplasmic marker) were detected. **e** mRNA expression of YAP target genes were measured with real-time PCR. **f** Protein levels of YAP and PAR2 were examined with western blot in stable transfectant HT-29 cells with or without shRNA against PAR2 (sh-PAR2). **g** Expression of F2RL1 (PAR2), YAP, and (**h**) its target genes were examined with real-time PCR in stable transfectant HT-29 cells. **i** p127-YAP, p397-YAP, and total-YAP were measured with western blot in stable transfectant HT-29 cells. *Note*: To have equal amount of YAP protein, the lysates of HT-29-vector and HT-29-shPAR2 are loaded at the ratio of 1:2.5. Data were shown as mean ± SEM. N.S. no significance; *p < 0.05; **p < 0.01; ***p < 0.001 vs. corresponding control

To investigate the role of PAR2 on YAP, the direct effect of PAR2 activation on YAP expression was tested. Firstly, it was found that both trypsin and PAR2-activating peptide (PAR2-AP) increased YAP expression at the protein level (Fig. 3a, b), but not at the mRNA level (Fig. 3c). The nuclear accumulation of YAP was observed at 0.5 h after treatment (Fig. 3d). Accordingly, CTGF and CYR61, the transcriptional target genes of YAP, were also elevated by trypsin and PAR2-AP (Fig. 3e). Secondly, since the autocrine loop of PAR2 and its activating proteases were observed in most colon cancer cells^9^, PAR2 was stably knocked down with shRNA in HT-29 and SW620 cells. Consistent with the findings in vivo, knockdown of PAR2 dramatically reduced the protein level of YAP, but not the mRNA level (Fig. 3f, g). The expression of target genes was also downregulated (Fig. 3h). Of note, neither activation nor knockdown of PAR2 has any significant effect on YAP mRNA. Emerging evidence shows that phosphorylation plays a critical role in the control of YAP activity and stability at the post-transcriptional level^1^. We found that PAR2 activation induced dephosphorylation of YAP at sites ser127 and ser397, which started at 30 min and recovered by 2 h (Fig. 3a, b). The same effect was also observed in other epithelial cell lines (Figure S3). Consistently, knockdown of PAR2 increased the phosphorylation of YAP in shPAR2 stable cells (Fig. 3i). The data suggested that activation of PAR2 signaling upregulates YAP protein expression through a phosphorylation-related manner in colonic epithelial cells.

### YAP rescue PAR2 deficiency-related apoptosis

**Fig. 4:**
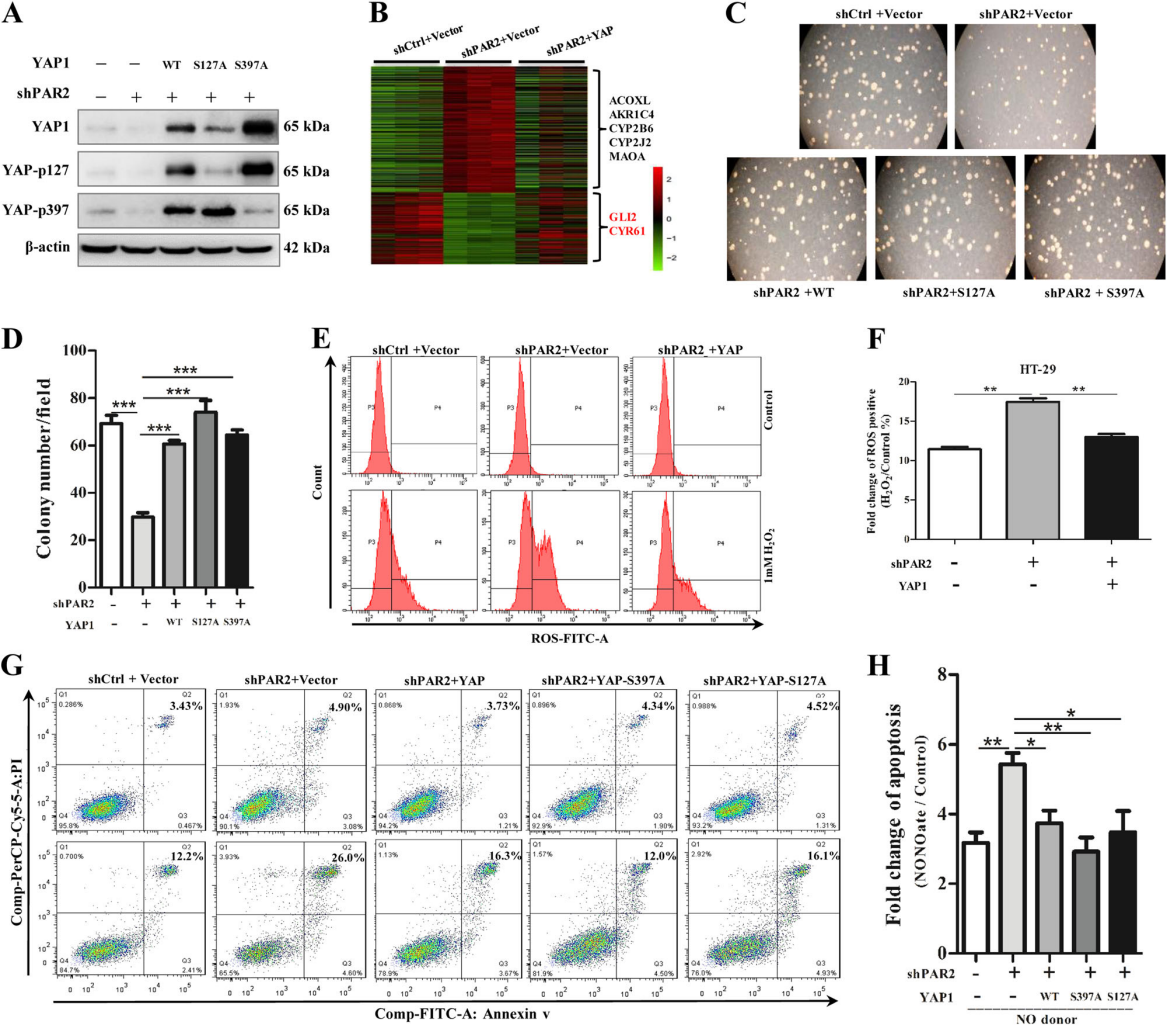
YAP rescues PAR2 deficiency-induced apoptosis and colony formation. Different forms of YAP (WT, S127A or S397A) were transfected into stable transfectant HT-29 cells with shRNA against PAR2. **a** The levels of p127-YAP, p397-YAP, and total-YAP were measured with western blot. **b** Heatmap showing the 464 rescued genes upon YAP replenishment (shPAR2 + YAP) in shPAR2 HT-29 cells (shPAR2 + vector). Some of the genes related to ROS production (black) and target genes of YAP (CYR61 and GLI2) (red) were listed. **c** and **d** HT-29–shPAR2 cells stably expressing YAP (WT), YAP-S127A, YAP-S397A, or vector were cultured in type I collagen and were analyzed for colony formation (5000 cells/well in 12-well plate). **c** Images of representative field (magnification, 250 ×). **d** Number of colony was quantified from five consecutive field of each group. **e** and **f** ROS was analyzed with flow cytometry in HT-29 stably transfect cells after treatment with H_2_O_2_ at 1 mM for 2 h. The relative change was obtained by H_2_O_2_ vs. control group. **g** and **h** Apoptosis was analyzed with Annexin V/PI staining assay in HT-29 stably transfect cells after treatment with NO donor DETA NONOate at 0.5 mM for 48 h. The fold change was obtained by NONOate vs. control samples. Data are shown as mean ± SEM. ***p < 0.001 vs. corresponding control

To understand the biological function of YAP in colonic mucosal damage, different isoforms of YAP1 were transfected to replenish the reduction of YAP induced by PAR2 knockdown (Fig. 4a, Figure S4a). Microarray analysis revealed that 464 genes were rescued with YAP replenishment in PAR2 knockdown cells (Fig. 4b). GO functional enrichment analysis showed that the rescued genes are strongly correlated with cell proliferation (Figure S4b). As expected, forced expression of YAP rescued the colony formation ability of HT-29 cells reduced by PAR2 inhibition in 2D and 3D culture (Fig. 4c, d, Figure S4c). Additionally, microarray date showed that some of the genes related to reactive oxygen species (ROS) production were downregulated by YAP replenishment, such as ACOXL, AKR1C4, CYP2B6, CYP2J2, and MAOA (Fig. 4b). Since ROS and reactive nitrogen intermediates (RNI) generated by inflammatory cells play critical roles in the pathogenesis of colitis^13,14^, we further tested the effects of PAR2 and YAP on H_2_O_2_-induced ROS generation. As shown, inactivation of PAR2 signaling dramatically sensitized colonic cells to H_2_O_2_-induced ROS generation (Fig. 4e, f) and NO donor-induced apoptosis (Fig. 4g, h). More importantly, replenishment of YAP significantly abolished the sensitivity to H_2_O_2_ and NO in PAR2-deficient cells (Fig. 4e-h). These results strongly suggest that PAR2 signaling protects colonic cells against oxidative stress at least partly through the stabilization of YAP.

### PAR2 activation stabilizes YAP protein through dephosphorylation independent of Lats1

**Fig. 5:**
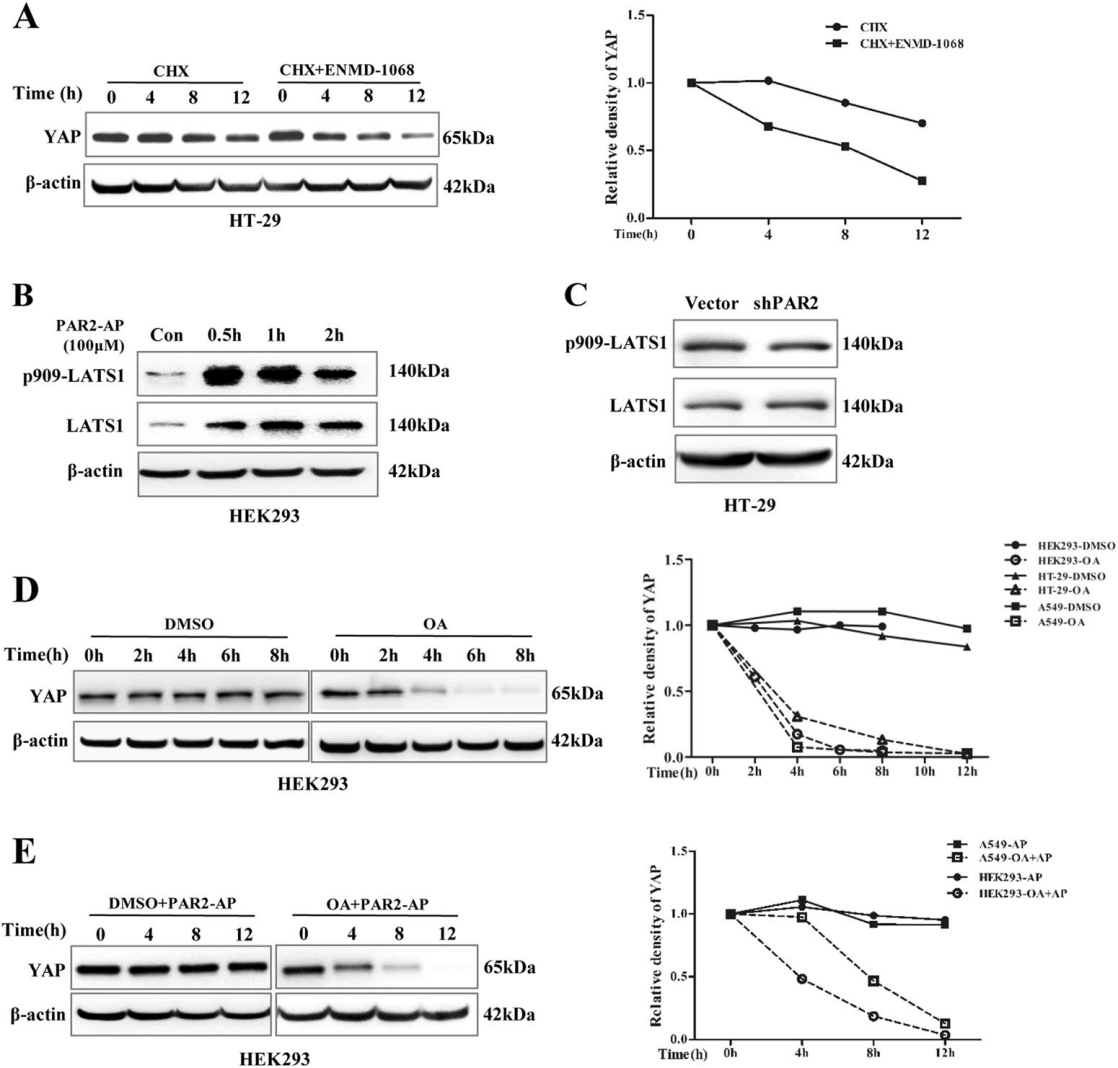
PAR2 activation induced dephosphorylation of YAP independent of Lats1. **a** HT-29 cells were pretreated with the inhibitor of protein synthesis, cycloheximide (CHX, 100 mg/ml). Then the cells were challenged with or without PAR2 inhibitor ENMD-1068 (1 mM) for different time periods. **b** HEK293 cells were serum-starved for 12 h and then stimulated with 100 μM PAR2-AP for different times. The levels of YAP, p909-Lats1, and Lats1 were measured with western blot. **c** Western blot was used to detect p909-Lats1 and Lats1 in HT-29–vector and HT-29–shPAR2 cells. **d** HEK293, A549, and HT-29 cells were pretreated with the inhibitor of protein synthesis, cycloheximide (CHX, 100 mg/ml), and then treated with OA (100 nM) for different time periods. Relative density of YAP from different cells was quantified (right). **e** A549 or HEK293 cells were pretreated with or without 100 nM OA and CHX, and then challenged with 100 µM PAR2-AP for different time periods. Relative density of YAP from different cells were quantified (right)

Since PAR2 did not show any effect on YAP mRNA, the stabilization of YAP protein was examined. It was found that inhibition of PAR2 with ENMD-1068 accelerated the degradation of YAP protein (Fig. 5a).

Lats kinase, a core component in the Hippo pathway, directly induces the phosphorylation and degradation of YAP^1^. To determine whether dephosphorylation of YAP by PAR2 is mediated by suppression of Lats kinase, we measured the phosphorylation of Lats1 at S909, a marker of Lats activation^15^. However, PAR2 activation increased rather than reduced the phosphorylation of Lats at S909 (Fig. 5b), neither shRNA against PAR2 induced the activation of Lats (Fig. 5c). In addition, they are all independent of cell density (data not shown). In order to strengthen our observations, a positive control was performed as reported previously^16^. Dephosphorylation of both Lats and YAP was induced by FBS (Figure S3c). It suggests that PAR2 activation stabilizes YAP and induces dephosphorylation of YAP, which is independent of Lats1 in colonic epithelial cells.

Phosphorylation status of protein is regulated via the balance between kinases and protein phosphatases. The phosphoprotein phosphatase superfamily members are major eukaryotic Ser/Thr protein phosphatases that are involved in a wide variety of biological processes^17^. To test whether PAR2 activation maintains the stability of YAP through protein phosphatases (PPs), the inhibitor okadaic acid (OA) was used. Interestingly, inhibition of PPs activity with OA significantly reduced the stability of YAP protein (Fig. 5d; Figure S5a). The stabilization of YAP by PAR2-AP was abolished by OA in different cell lines (Fig. 5f, Figure S5b). These data suggest that PAR2 signaling stabilizes YAP protein through OA-sensitive PPs.

### PAR2 activation enhances the interaction of YAP with PP1

**Fig. 6:**
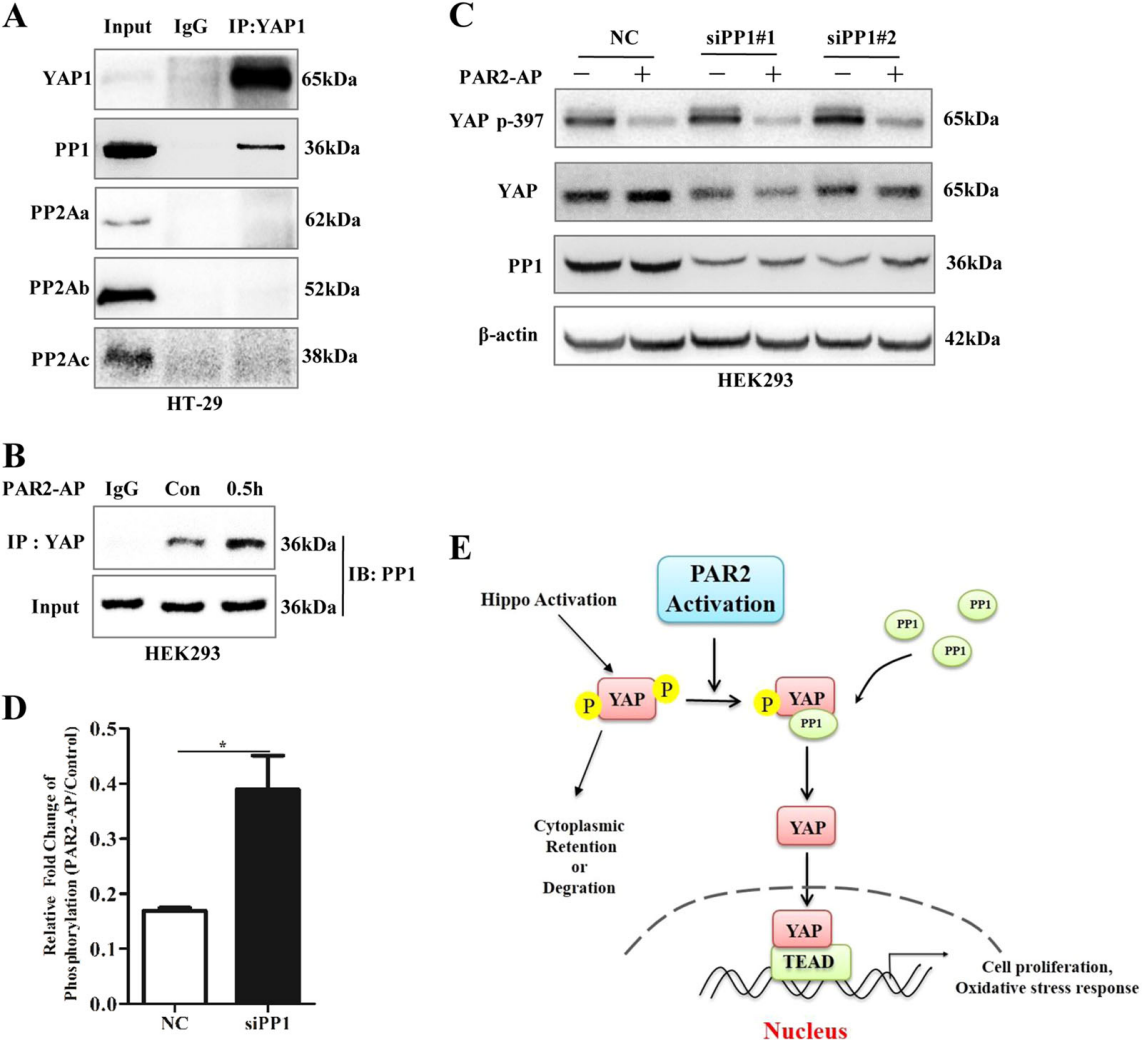
PP1-mediated PAR2 activation induced dephosphorylation of YAP. **a** Whole-cell lysate of HT-29 was immunoprecipitated with anti-YAP antibody or control IgG, followed by western blot for PP1, PP2a, PP2Ab, and PP2Ac. **b** HEK293 cells were treated with PAR2-AP for 30 min. Then, cell lysates were collected and used for immunoprecipitation with anti-YAP antibody. PP1 were detected by western blot. **c** Twenty-four hours after transfection with two different siRNAs against PP1, HEK293 cells were serum-starved for 12 h and then stimulated with PAR2-AP for 30 min. Western blot was used to determine p397-YAP and YAP. **d** The density of WB bands from panel **d** was analyzed by image-pro-plus soft system. Fold change of YAP phosphorylation is calculated as [p397-YAP /YAP]_PAR2-AP_/[p397-YAP /YAP]_NC_. Data were shown as mean ± SEM. *p < 0.05 vs. NC control. **e** Diagram of signaling pathways responsible for PAR2-regulated YAP stability and function

It has been reported that protein phosphatases PP1 and PP2A interact with YAP to regulate its phosphorylation and activity in various cells^18,19^. We hypothesized that PAR2 regulates the phosphorylation of YAP through enhancing the interaction between YAP and PP1 or PP2A. Immunoprecipitation data showed that endogenous PP1, but not PP2A, form a protein complex with YAP in different cell lines tested (Fig. 6a; Figure S6a). In addition, activation of PAR2 with AP increased the binding of YAP and PP1 (Fig. 6b; Figure S6b). Moreover, inhibition of PP1 with different siRNA (Fig. 6c) not only significantly inhibited the upregulation of YAP protein, but also partly blocked dephosphorylation of YAP induced by PAR2 (Fig. 6d; Figure S6e). Since the importance of ERK signaling after PAR2 activation, the role of ERK on YAP stabilization was tested. Inhibitors of ERK did not abolish the inhibitory effect of PAR2 activation on YAP phosphorylation at ser397 (Figure S7). All these suggest that PAR2 activation stabilizes YAP protein through a PP1-mediated dephosphorylation pathway (Fig. 6e).

## Discussion

The mechanisms underlying mucosal regeneration in the colon after chronic injury and inflammation are central questions in our understanding of the complex processes of homeostasis and tumorigenesis. Most recently, an increasing body of evidence implies that YAP-dependent signaling controls epithelium regeneration response to injury in the gut^8,20,21^. YAP is dispensable for normal intestinal homeostasis, but is required for intestinal regeneration following injury^8^. However, the link between mucosal damage and YAP signaling is absent. Here we show that PAR2 signaling mediates colonic mucosal regeneration through the stabilization of YAP protein in DSS-induced colitis in mouse. Actually, the regulation of YAP by PARs (both PAR1 and PAR2) has recently been demonstrated^22^. The mechanism of PAR2-induced YAP activation remains unknown, although PAR1 activates YAP through the inhibition of Lats1/2 kinase by G_12/13_ and Rho GTPase^22^. In current study, our findings suggest that Lats is not involved in the dephosphorylation of YAP induced by PAR2. However, YAP protein is stabilized by PAR2 through PP1-dependent dephosphorylation. Furthermore, our findings reveal that PAR2 signaling mediates colonic mucosal regeneration through stabilization of YAP in colitis model.

The role of PAR2 in colitis is contradictory. It has been shown that PAR2 and its agonists play a proinflammatory and damaging role in colitis models. For instance, antagonism or knockout of PAR2 protects against colitis in different models^23,24^. It is also found that PAR2 agonists induce adverse effects on the colon of mice^25^. Although cytokine expression was reduced in the PAR2-KO mouse colons, in current study, we demonstrated that PAR2 activation plays prosurvival roles on colonic epithelium through stabilization of YAP in colitis model. The deficiency of PAR2 signaling delayed the recovery of crypt architecture and mucosal regeneration after DSS-induced colitis in mice. Our findings are consistent with recent reports that activation of PAR2 is crucial for normal regeneration in different tissues^7^. However, the mechanism by which PAR2 modulates tissue regeneration is not clear. After pancreatitis, PAR2 participates in the regeneration of pancreas through the regulation of β-cell trans-differentiation and apoptosis^7^. Here, we demonstrated that PAR2 enhances YAP signaling and increases the survival of colonic cell under the stress induced by nitric oxide. In accord with our findings, it has been reported that PAR2-treated normal cells were more resistant to serum starvation-induced stress^26^. Moreover, our date indicated that the prosurvival effect of PAR2 signaling might be due to YAP-dependent regulation of oxidative stress response. Recently, it has been shown that YAP interacted with FoxO1 and promoted antioxidant genes expression in cardiomyocytes, including catalase and manganese superoxide dismutase (MnSOD)^27^. Although the change of antioxidant genes was not observed, we demonstrated that YAP repressed the expression of a gene set related to ROS production. These may explain the abolishment of ROS accumulation by YAP replenishment in PAR2-knockout cells. Thus, it provides a novel mechanism by which PAR2 signaling protect epithelial cells against oxidative stress in the colon.

Interestingly, serine proteases have been implicated in crypt regeneration in the colon. The activity of serine protease in vivo is balanced by endogenous inhibitors, the serpins (serine protease inhibitors), which are highly conserved protein superfamily involved in tissue injury and inflammation^28,29^. Serpine1 (Serpin E1), a member of the serpin superfamily, has been shown to mediate the fission of crypt and Wnt5a-dependent colonic crypt regeneration after injury^30^. Notably, Serpine1 is also known as plasminogen activator inhibitor (PAI) and is shown to inhibit trypsin activity^31^. In addition, as the receptor of thrombin, PAR1 signaling mediates endothelial barrier breakdown and controls endothelial barrier integrity^32^. However, the effect of PAR1 on homeostasis, regrowth, and repair of the gut mucosa has been scarcely addressed. In common with PAR2, PAR1 has been shown to enhance the YAP activity in vitro^22^. However, studies in mice have demonstrated that PAR1 activation induces epithelial apoptosis and increases intestinal permeability^33^. Most recently, it was shown with 3-D culture of human colon organoid that thrombin through PAR1 and PAR4 favors the maturation of colon epithelial cells, while it reduces their regenerative capacities^34^. It seems like PAR1 and PAR2 may have differential functions on mucosal homeostasis and repair. In the present study, our findings indicate that PAR2 signaling plays an important role in regulating colonic mucosal regeneration, although the mechanisms involved in this process remain unknown.

Considering the function of PAR2 as a sensor of extracellular serine protease, the origin of endogenous protease, which activates PAR2 and regulates tissue regeneration, after tissue damage is a key question. PAR2 signaling can be selectively activated by various endogenous serine proteinases, which are widely involved in pathogenesis in the gut^5^. PAR2-activating proteases are abundant in the gut lumen, such as trypsin secreted primarily by the pancreas^35^, as well as trypsin-like enzymes produced by the bacteria^36^. Immune cells release various PAR2-activating proteases, such as tryptase and elastase, released by mast cells or neutrophils^37^. Coagulation involves the activation of coagulation factor V, XII and KLKs, all of which have been shown to activate PAR2^38^. Thus, it is possible that PAR2 signaling is triggered by disruption of the barrier function, infiltration of immune cells, and tissue damage, thus leading to the unusual exposure of epithelial cells to serine proteases. Through the stabilization of YAP protein, PAR2 signaling promotes cell survival under stressful environment. Similarly, the amount of extracellular proteases decreases gradually with the recovery of barrier function and resolving of inflammation. However, whether immune cells or integrity of barrier regulates colonic tissue regeneration process and whether it is mediated by PAR2 signaling, all these need further investigation in the future.

Taken together, our results present new evidence showing a novel mechanism by which YAP is stabilized by PAR2 signaling, which can be activated selectively by extracellular protease. Deficiency of PAR2 impairs colonic mucosal regeneration after DSS-induced injury by inhibiting YAP expression, thus suggesting the importance of PAR2 signaling in the control of mucosal regeneration in the colon.

## Electronic supplementary material

Suppl Figure S1

Suppl Figure S2

Suppl Figure S3

Suppl Figure S4

Suppl Figure S5

Suppl Figure S6

Suppl Figure S6 legend

Suppl Figure S7

Suppl Table 1
