# Supplementary figures and images for: Protease-activated receptor 2 signaling modulates susceptibility of colonic epithelium to injury through stabilization of YAP in vivo
Source: Cell Death Dis. 2018 Sep 20;9(10):949. doi: 10.1038/s41419-018-0995-x (PMC6148223; doi:10.1038/s41419-018-0995-x)

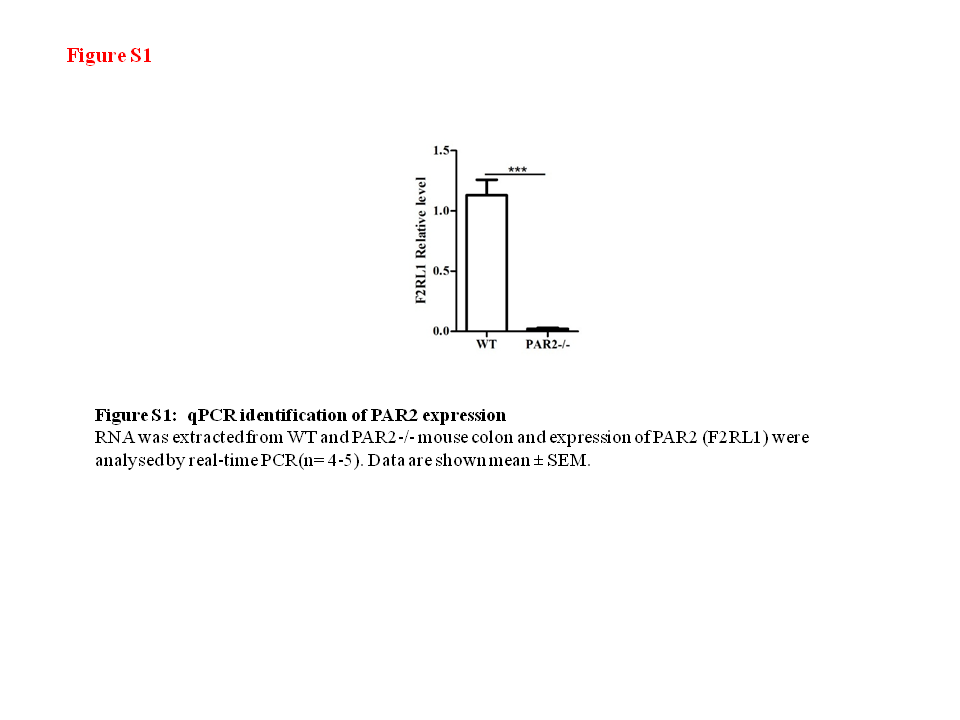

Supplement: Supplementary file 1 — Suppl Figure S1 [file 41419_2018_995_MOESM1_ESM.tif]

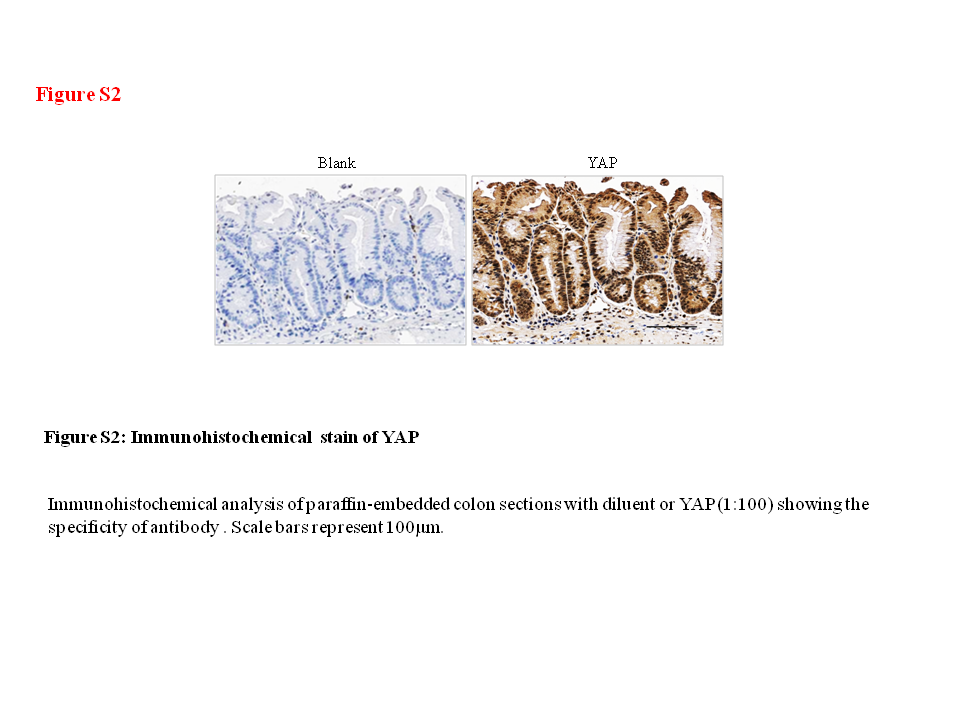

Supplement: Supplementary file 2 — Suppl Figure S2 [file 41419_2018_995_MOESM2_ESM.tif]

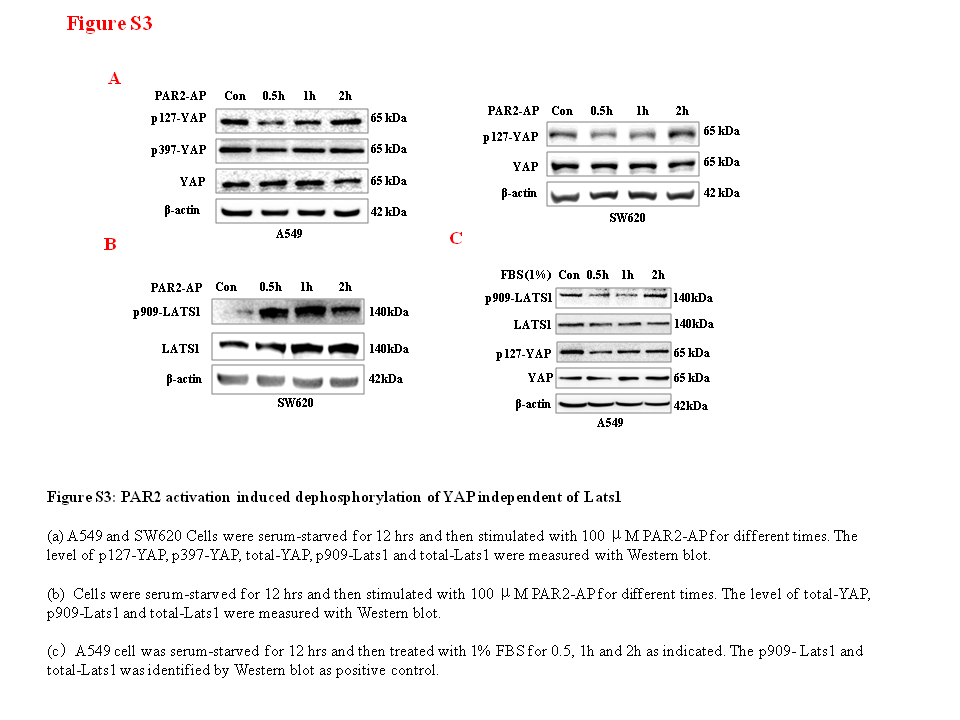

Supplement: Supplementary file 3 — Suppl Figure S3 [file 41419_2018_995_MOESM3_ESM.tif]

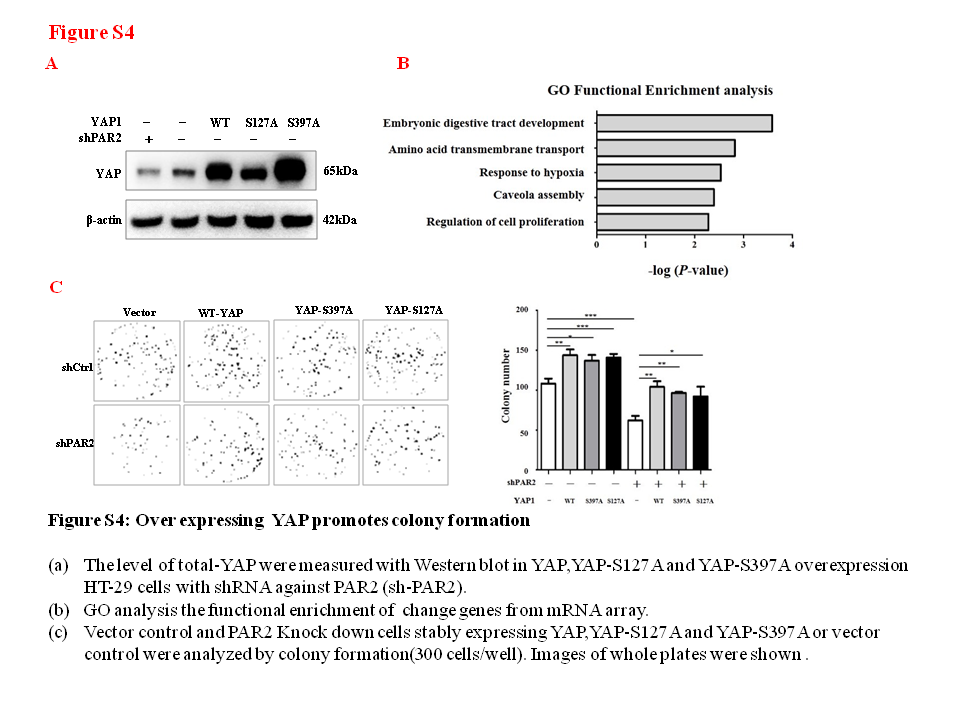

Supplement: Supplementary file 4 — Suppl Figure S4 [file 41419_2018_995_MOESM4_ESM.tif]

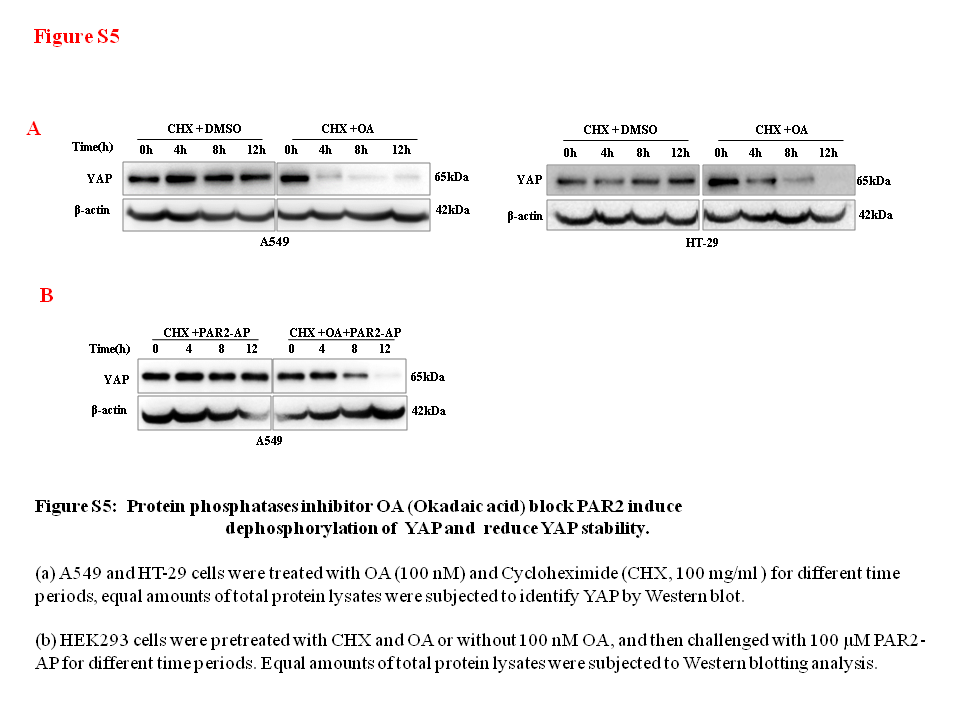

Supplement: Supplementary file 5 — Suppl Figure S5 [file 41419_2018_995_MOESM5_ESM.tif]

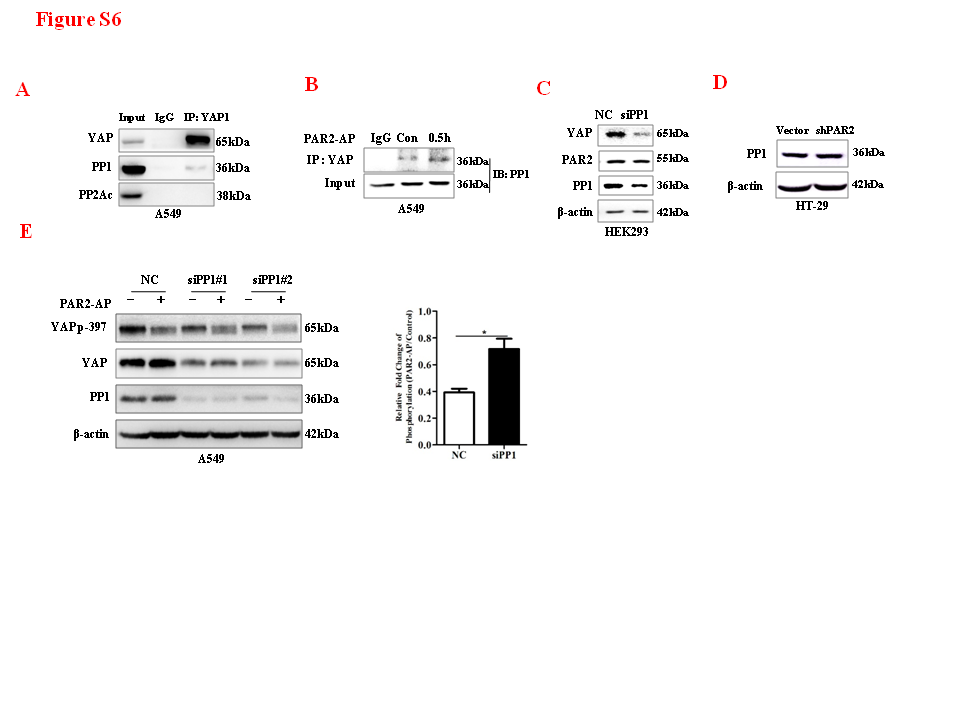

Supplement: Supplementary file 6 — Suppl Figure S6 [file 41419_2018_995_MOESM6_ESM.tif]

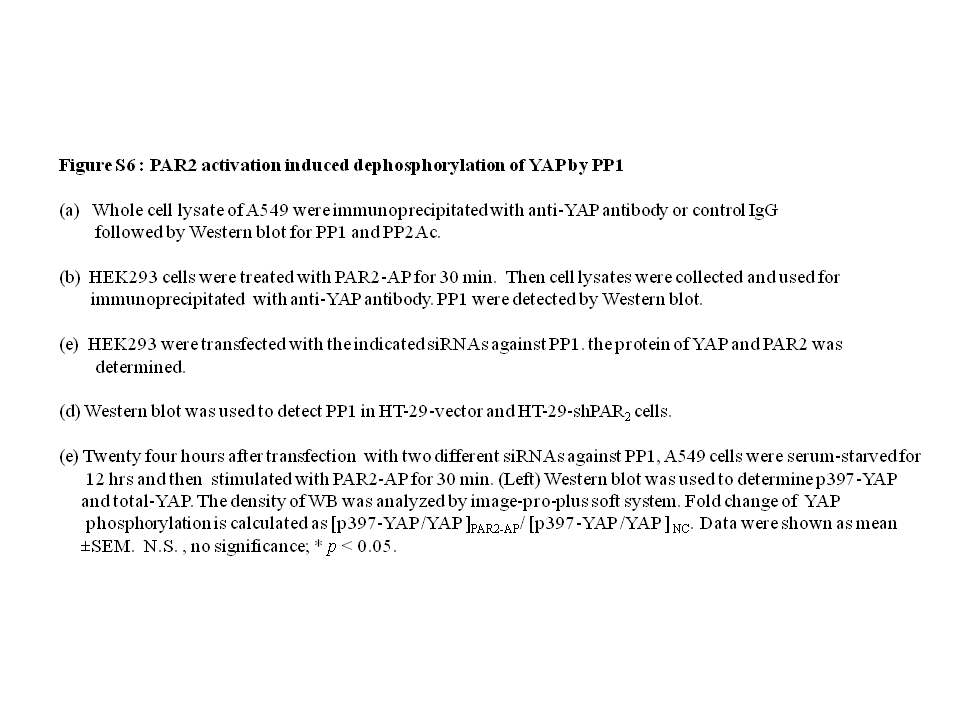

Supplement: Supplementary file 7 — Suppl Figure S6 legend [file 41419_2018_995_MOESM7_ESM.tif]

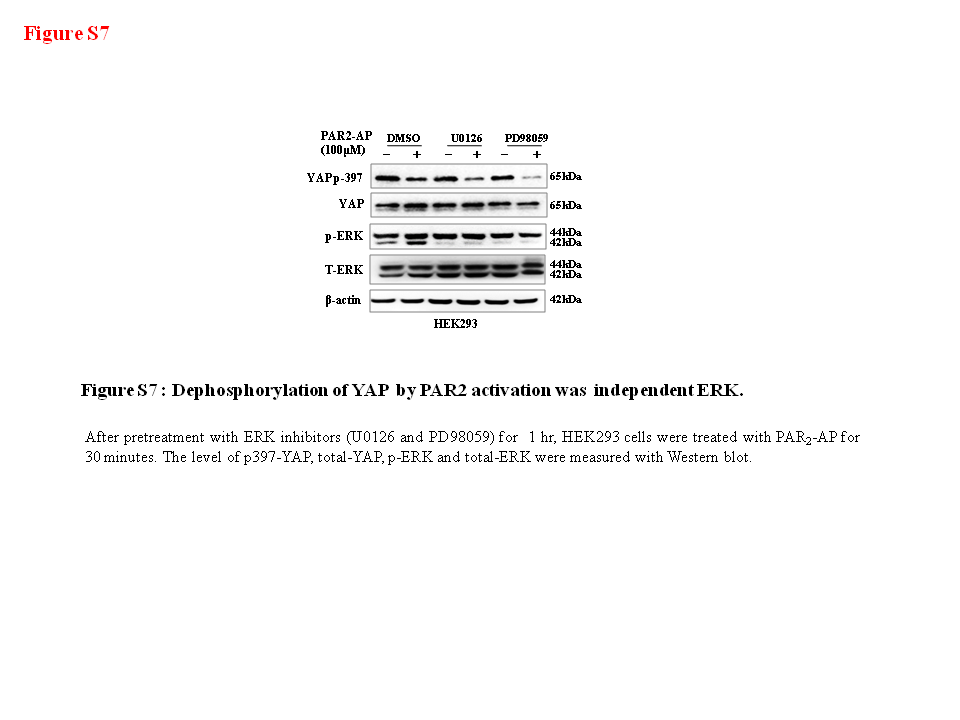

Supplement: Supplementary file 8 — Suppl Figure S7 [file 41419_2018_995_MOESM8_ESM.tif]

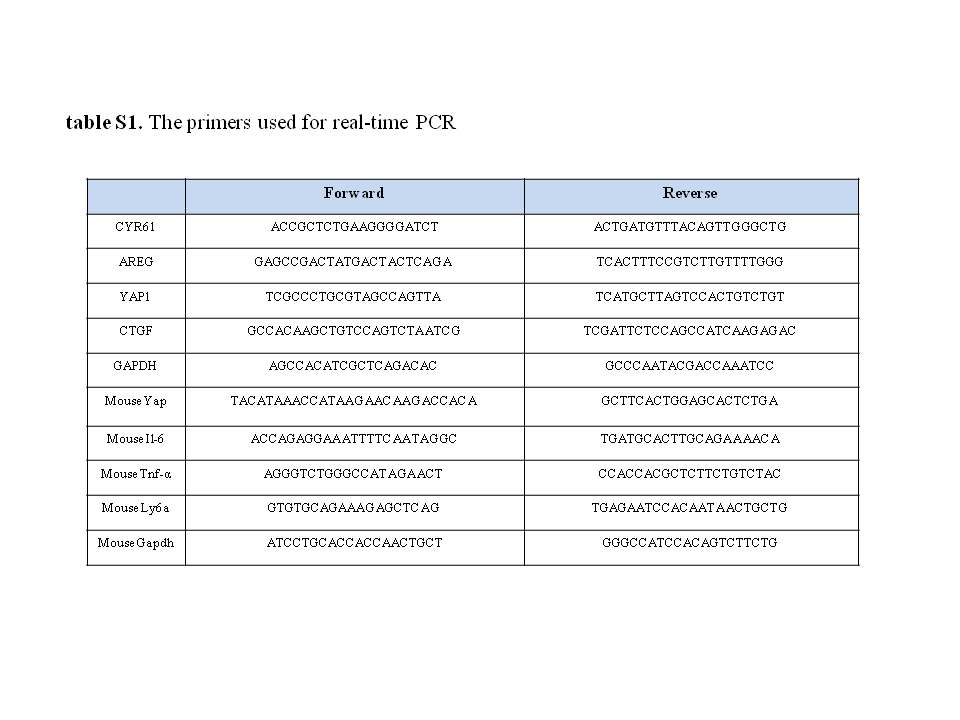

Supplement: Supplementary file 9 — Suppl Table 1 [file 41419_2018_995_MOESM9_ESM.tif]
